# Multiple Arterial Grafting During Coronary Artery Bypass Graft Surgery in Diabetic and Non-Diabetic Patients: A Short- and Long-Term Analysis at a Single Center †

**DOI:** 10.3390/jcm13237082

**Published:** 2024-11-23

**Authors:** Miralem Jasarevic, Oskar Krueger, Jan Strathmann, Marinela Jasarevic, Sharaf-Eldin Shehada, Jarowit Adam Piotrowski, Parwis Massoudy, Heinz Jakob, Markus Kamler, Payam Akhyari, Matthias Thielmann

**Affiliations:** 1 Department of Thoracic and Cardiovascular Surgery, West German Heart and Vascular Center, Hufelandstrasse 55, 45147 Essen, Germany; miralem.jasarevic@hotmail.com (M.J.); oskar.krueger@uk-essen.de (O.K.); jan.strathmann@uk-essen.de (J.S.); marinela.jasarevic@uk-essen.de (M.J.); sharaf-eldin.shehada@uk-essen.de (S.-E.S.); heinz.jakob@uk-essen.de (H.J.); markus.kamler@uk-essen.de (M.K.); payam.akhyari@uk-essen.de (P.A.); 2 Department of Cardiac, Thoracic and Vascular Surgery, St. Johannes Hospital Dortmund, Johannesstraße 9-17, 44137 Dortmund, Germany; jarowit.piotrowski@joho-dortmund.de; 3 Department of Cardiac Surgery, Hospital Passau, Innstraße 76, 94032 Passau, Germany; parwis.massoudy@klinikum-passau.de

**Keywords:** coronary artery bypass surgery, multiple arterial grafting, diabetes mellitus

## Abstract

**Background/Objectives**: Coronary artery bypass surgery (CABG) with multiple arterial grafting (MAG) has shown potential to improve patient survival compared to single arterial bypass grafting. Whether this superiority in survival also exists in diabetics is uncertain. We therefore aimed to compare short and long-term outcomes of MAG in diabetic versus non-diabetic patients. **Methods**: In this retrospective study, we investigated short- and long-term clinical outcomes of diabetic (*n* = 256) and non-diabetic (*n* = 800) patients undergoing CABG with MAG between January 1999 and December 2019 at our institution. **Results**: Diabetics had a significantly higher EuroScore II (1.37 ± 2.4 vs. 0.88 ± 1.58, *p* < 0.0001) and underwent significantly less bilateral internal thoracic artery (BITA) grafting (51.95% vs. 67.75%; *p* < 0.0001) compared to non-diabetics. The incidence of postoperative adverse events, such as pneumonia, stroke, and sepsis, did not differ between the two groups. However, diabetics suffered significantly more often from post-cardiotomy cardiogenic shock, renal failure requiring dialysis, and sternal wound infections over the entire follow-up period. Non-diabetics had a significantly higher median survival time of 19.6 years compared to 14.54 years found in diabetic patients (*p* < 0.0001). **Conclusions**: Among patients undergoing MAG, diabetic individuals were found to have a significantly lower overall median survival. This emphasizes the importance of diabetes as a risk factor in choosing individual surgical strategies.

## 1. Introduction

Coronary artery disease (CAD) is the leading cause of morbidity and mortality globally [1], and coronary artery bypass grafting (CABG) is the recommended treatment for patients with complex multivessel coronary artery disease [2]. The use of multiple arterial grafts (MAG), particularly using the internal thoracic arteries (ITA), has been shown to enhance long-term patency and survival outcomes compared to single arterial grafts or venous grafts [3,4,5,6]. However, the presence of diabetes mellitus—a known risk factor for poor outcomes—raises concerns about the efficacy and safety of MAG in this patient group.

Diabetes mellitus is associated with a host of complications that can influence both short- and long-term outcomes following CABG, including increased rates of inflammation, endothelial dysfunction, and impaired wound healing. These biological mechanisms contribute to higher risks of perioperative morbidity and long-term complications [7,8,9]. The current study was designed to explicitly compare the outcomes of diabetic versus non-diabetic patients undergoing CABG with MAG over a 20-year period at a single center. The primary objective is to evaluate whether diabetic patients undergoing MAG experience higher perioperative complications and reduced long-term survival compared to non-diabetic patients, thus contributing to a better understanding of the risks associated with diabetes in surgical outcomes.

We hypothesize that diabetic patients undergoing MAG would exhibit higher perioperative complications and reduced long-term survival compared to non-diabetic patients. By identifying and understanding these mechanisms, the research aims to support the development of tailored treatment strategies that could enhance surgical outcomes and long-term recovery in this high-risk population.

## 2. Materials and Methods

### 2.1. Study Design and Population

This retrospective cohort study analyzed the records of 1056 consecutive patients who underwent CABG with MAG, at the West German Heart and Vascular Center, Essen, between January 1999 and December 2019. The patient cohort was divided into two groups based on the presence of diabetes mellitus: diabetic (Group I; *n* = 256) and non-diabetic (Group II; *n* = 800) patients. Diabetic status was determined based on preoperative medical records, including fasting glucose levels, HbA1c measurements, and the use of insulin or oral antidiabetic medication.

### 2.2. Study Endpoints

The primary endpoint of the study was 30-day mortality, defined as death from operation-related causes within 30 days post-surgery. Secondary endpoints included postoperative complications and long-term survival. Postoperative complications included the incidence of post-cardiotomy cardiogenic shock (PCCS), renal failure requiring dialysis, stroke, myocardial infarction, pneumonia, sepsis, and sternal wound infections.

### 2.3. Surgical Procedure

All patients underwent standard CABG procedures involving the use of multiple arterial grafts, defined as the use of ≥2 arterial grafts, primarily the left and/or right internal thoracic arteries (LITA, RITA). Within the analyzed cohort, the predominant preparation technique for the internal thoracic artery was the pedicle technique, used in 469 of 591 cases (79.36%). The skeletonization technique was performed in 67 of 591 cases (11.34%), and a combination of both techniques was used in 55 of 591 cases (9.31%). In terms of surgical approach, off-pump coronary artery bypass (OPCAB) was conducted in 16 of 1050 cases (1.52%), while the vast majority underwent on-pump coronary artery bypass (ONCAB), performed in 1034 of 1050 cases (98.48%).

Bilateral internal thoracic artery (BITA) grafting was performed in patients where it was feasible, those who were at lower risk for sternal wound infections and younger than 70 years old, while additional arterial or venous grafts were used as necessary. The decision to use BITA or other arterial conduits was at the discretion of the operating surgeon, based on the patient’s coronary anatomy, comorbidities, and intraoperative findings.

### 2.4. Data Collection

Data collection was conducted retrospectively using the database of the West German Heart and Vascular Center in Essen. Initially, all treatment protocols for patients who underwent surgery between 1999 and 2019 (>25,000 patients) were manually reviewed, and 1056 patients who received CABG using the MAG technique were identified. The medical history forms, physician letters, operative reports, and transfer and discharge letters of these patients were analyzed. Data included preoperative demographics (age, gender, Body mass index, and comorbidities), intraoperative variables (type and number of grafts, cross-clamp time, and cardiopulmonary bypass time), and postoperative outcomes. Long-term survival and mortality data were obtained from follow-up records, national death registries, and through contacting the relevant civil registry offices. The follow-up rate of 93% for determining long-term survival is satisfactory. However, it should be noted that the follow-up duration varies, with long-term survival data of ≥10 years dating back to surgeries performed between 1999 and 2009.

### 2.5. Statistical Analysis

Descriptive statistics were compiled using Microsoft Excel 2022 (Microsoft Corporation, Redmond, WA, USA) with continuous variables expressed as mean ± standard deviation and categorical variables as absolute numbers with corresponding percentages. For comparisons and long-term survival analysis, GraphPad Prism version 9.4.1 (GraphPad Software, San Diego, CA, USA) was used. *P*-values for continuous variables were calculated using unpaired parametric T-tests, and chi-square tests were used for categorical variables. A significance threshold was set at *p* < 0.05. Kaplan–Meier curves were generated for survival analysis, with log-rank tests used to compare survival curves and calculate *p*-values and hazard ratios. Visualizations of the results were produced using both Microsoft Excel 2022 (Microsoft Corporation, Redmond, WA, USA) and GraphPad Prism version 9.4.1 GraphPad Software, San Diego, CA, USA).

## 3. Results

### 3.1. Patient Demographics and Preoperative Characteristics

The average age of the diabetic group was 65.94 ± 9.35 years, while the non-diabetic group had an average age of 62.57 ± 9.99 years (*p* < 0.0001). The proportion of women was higher among diabetics, with 28.13% (*n* = 72), compared to 19.75% (*n* = 158) in the non-diabetic group (*p* = 0.005). There was a significant difference in body mass index (BMI) between the two groups. The average BMI in the diabetic group was 29.05 ± 4.53, whereas it was 27.34 ± 3.89 in the non-diabetic group (*p* < 0.0001). NYHA I and II were each observed in 32% of patients, while NYHA III was present in 30% and NYHA IV in 6% of patients. All comorbidities and risk factors, except for family history, hyperlipoproteinemia, and smoking, were more prevalent in the diabetic group. The distribution of comorbidities and risk factors in both groups is illustrated in Table 1.

The mean EuroScore II was significantly higher in diabetic patients (1.37 ± 2.4 vs. 0.88 ± 1.58, *p* < 0.0001), reflecting a greater operative risk. The proportion of patients undergoing BITA grafting was lower in diabetics (51.95% vs. 67.75%, *p* < 0.0001), potentially due to concerns about sternal wound healing.

There were significant differences between the two groups regarding the extent of coronary artery disease. In the diabetic group, 72.66% (*n* = 186) had triple-vessel disease without accompanying left main stenosis, compared to 58.45% (*n* = 467) in the non-diabetic group (*p* < 0.0001). Conversely, both double-vessel disease with left main stenosis and triple-vessel disease with left main stenosis were more common in the non-diabetic group, at 8.39% (*n* = 67) and 22.65% (*n* = 181), respectively, compared to 3.91% (*n* = 10, *p* = 0.02) and 16.8% (*n* = 43, *p* = 0.05) in the diabetic group. Additionally, left main stenosis was present in 20.7% (*n* = 53) of diabetics and in 31.0% (*n* = 248) of non-diabetics (*p* = 0.002).

Table 2 shows the distribution of left ventricular ejection fraction (LVEF) between diabetic and non-diabetic patients, highlighting the poorer preoperative cardiac function in diabetics.

### 3.2. Intraoperative Findings

Mean number of arterial grafts used was similar between the two groups, with diabetic patients receiving an average of 3.27 ± 0.96 grafts, compared to 3.32 ± 1.02 grafts in non-diabetic patients (*p* = 0.46). The mean cardiopulmonary bypass time was slightly longer in diabetics, at 110.29 ± 41.57 min compared to 107.17 ± 35.07 min in non-diabetics (*p* = 0.25). The aortic cross-clamp times were also comparable between the two groups (75.49 ± 27.3 min in diabetics vs. 75.79 ± 25.11 min in non-diabetics, *p* = 0.87). Diabetics had a significantly longer reperfusion time (26.04 ± 20.83 min vs. 22.66 ± 11.62 min, *p* = 0.008). The mean operative time was slightly longer in the diabetic group, at 258.02 ± 67.02 min, compared to 262.65 ± 64.64 min in non-diabetics (*p* = 0.32). Additionally, diabetic patients had a marginally lower mean arterial bypass flow (111.44 ± 57.02 mL/min) compared to non-diabetics (119.84 ± 61.97 mL/min, *p* = 0.06), though this difference was not statistically significant. The venous bypass flow rates were also comparable between groups, with diabetic patients averaging 65.06 ± 44.51 mL/min versus 69.59 ± 41.85 mL/min in non-diabetics (*p* = 0.52). The total bypass flow was slightly lower in diabetic patients (121.44 ± 68.84 mL/min) compared to non-diabetics (134.23 ± 78.23 mL/min, *p* = 0.07), although this difference was not statistically significant.

Significant differences were observed in the choice of arterial bypass grafts as shown in Table 3. In diabetics, total arterial BITA grafting was performed in 51.95% of patients (*n* = 133), whereas this was the case in 67.75% (*n* = 542, *p* < 0.0001) of patients in non-diabetics. Conversely, total arterial revascularization using one ITA and one RA (radial artery) was more frequently performed in the diabetic group, at 24.61% (*n* = 63), compared to 9.5% (*n* = 76, *p* < 0.0001) in the non-diabetic group.

Intraoperative complications included bleeding in 2.4% (*n* = 6) of diabetics, compared to 2.32% (*n* = 18) in non-diabetics. Flow-related problems occurred in 1.6% (*n* = 4) of diabetic patients and 2.32% (*n* = 18) in non-diabetics. Intraoperative reanimation occurred in 0% (*n* = 0) of diabetics and 0.39% (*n* = 3) of non-diabetics. A second run of heart-lung-machine was necessary for 1.24% (*n* = 3) of diabetics and 1.9% (*n* = 14) of non-diabetics.

### 3.3. Postoperative Findings

Total hospital stay, postoperative stay, and intensive care unit stay were longer in the diabetic group compared to the non-diabetic group, and these differences were statistically significant as shown in Table 4.

Diabetics experienced a higher incidence of several postoperative complications. Post-cardiotomy cardiogenic shock was significantly more common in diabetics (4.0% vs. 0.6%, *p* < 0.0001), as was renal failure requiring dialysis (12.4% vs. 5.2%, *p* < 0.0001). Sternal wound infections were also more frequent in diabetics (13.8% vs. 9.3%, *p* = 0.04). Reintubation was required in 13.1% of diabetic patients (*n* = 33), whereas it was necessary in only 6.26% of cases in the non-diabetic group (*n* = 49, *p* = 0.0005). Mechanical circulatory support using an intra-aortic balloon pump (IABP) was necessary in 8.33% of diabetic patients (*n* = 21) and 4.47% of non-diabetic patients (*n* = 35, *p* = 0.02). Revision bypass surgery was performed in 2.34% of diabetic patients (*n* = 6) and 0.63% of non-diabetic patients (*n* = 5, *p* = 0.02). The two groups showed significant differences in the average drainage volume during the first 24 h postoperatively. The average volume in diabetics was 877.24 ± 395.16 mL, compared to 1108.82 ± 643.61 mL in non-diabetics (*p* < 0.0001).

In diabetics, 13.78% of patients (*n* = 35) experienced sternal wound healing complications, compared to 9.31% (*n* = 74) in non-diabetics (*p* = 0.04). During the hospital stay, sternal dehiscence occurred in 2.52% of diabetic patients (*n* = 10), while it was observed in 1.54% of cases (*n* = 12, *p* = 0.02) in the non-diabetic group.

Postoperative complications were consistently more frequent in the diabetic group. The distribution of postoperative complications is shown in Table 5.

The 30-day mortality rate was marginally higher in diabetic patients (4.3% vs. 3.8%, *p* = 0.32), though this difference was not statistically significant (see Table 6).

Non-diabetic group shows better long-term survival outcomes compared to the diabetic group (see Table 7).

**Figure 1:**
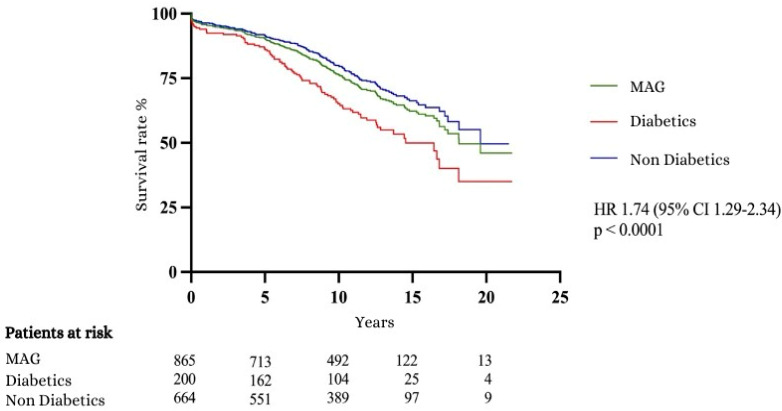
Kaplan–Meier curve 1: survival curves.

The following Kaplan–Meier curve (see Figure 1) illustrates the long-term survival comparison between diabetic and non-diabetic patients, demonstrating that diabetic patients experience reduced survival over the years. The statistical significance of these survival differences was evaluated using the log-rank test, affirming the validity of the observed disparities (HR 1.74, 95% CI 1.29–2.34, *p* < 0.0001).

## 4. Discussion

The findings of this study shed light on the complex relationship between diabetes mellitus and coronary artery bypass grafting with the use of multiple arterial grafting. Despite significant advancements in the treatment of diabetes mellitus, coronary artery disease remains a common comorbidity among these patients and contributes substantially to mortality. Patients with diabetes mellitus account for approximately 25–30% of those undergoing myocardial revascularization [10], with some studies reporting a prevalence as high as 45% in certain cohorts [11]. In the present study, 24.24% of the total patient group had diabetes. In the Essen cohort, 7.86% of the patients were diagnosed with insulin-dependent diabetes mellitus.

A comparison of the preoperative data between diabetic and non-diabetic patients highlights the vascular risk posed by diabetes mellitus. Peripheral arterial disease was present in 19.92% of diabetic patients but only in 12.13% of non-diabetics. The groups also showed significant differences regarding the extent of coronary artery disease, further confirming that diabetes mellitus is a risk factor for more severe stenosis in CAD. Furthermore, chronic kidney disease was more prevalent in the diabetic group. In contrast, diabetics exhibited a much higher prevalence of triple-vessel disease (72.66%) compared to non-diabetics (58.45%). Diabetic patients are at higher risk not only for CAD but specifically for multi-vessel disease, which is associated with reduced life expectancy [12].

Consistent with other studies [13], the Essen cohort also showed that additional risk factors and comorbidities, such as COPD (12.89%), carotid artery disease (17.97%), PAD (19.92%), arterial hypertension (91.02%), BMI ≥ 30 (36.33%), and renal disease (4.76%), were more prevalent in the diabetic group. These factors contributed to the higher EuroSCORE II values observed in diabetics (mean 1.37) compared to non-diabetics (mean 0.88). Notably, but in line with the literature [12,13], the diabetic group had a significantly higher proportion of women (28.2% vs. 19.75% in the non-diabetic group). Since women tend to develop CAD at an older age, this also explains the approximately 3.5-year higher average age of diabetics (65.94 years).

One of the key takeaways is the significantly higher rate of postoperative complications in diabetic patients. Post-cardiotomy cardiogenic shock, renal failure requiring dialysis, and sternal wound infections were all more common in the diabetic cohort. Increased incidence of PCSS in diabetics may be due to the combined effects of diabetic cardiomyopathy and the heightened vulnerability of these patients to perioperative stressors. Diabetic cardiomyopathy is known to compromise cardiac function, which may explain the higher rates of PCSS observed in this population [14].

Renal failure requiring dialysis was another significant complication in diabetic patients. This comes as no surprise, as preoperatively the prevalence of chronic kidney disease was significantly higher in the diabetic cohort. This aligns with the known susceptibility of diabetic patients to kidney damage [15]. Increased rates of renal failure underscore the importance of perioperative renal protection strategies in all patients undergoing CABG with MAG, with a particular focus on those with diabetes.

Diabetics are also at an increased risk of wound healing complications, as highlighted by the data from Essen, where sternal wound healing disorders were observed in 13.78% of diabetic patients, compared to 9.31% of non-diabetics. During hospital stay sternal dehiscence occurred in 2.52% of diabetic patients, more than 60% higher than in non-diabetics (1.54%). This complication is particularly important because it may negatively affect long-term outcomes by increasing hospital stays and morbidity; furthermore, it also significantly contributes to treatment costs. Known risks of poor wound healing in diabetics have influenced the surgical approach adopted by the surgeons in Essen. Using both mammary arteries for bypass construction involves more extensive chest surgery and, therefore, increases the risk of wound healing issues. As a result, only 51.95% of diabetics received total arterial BITA grafting, compared to 67.75% of non-diabetics.

While diabetic patients had more complications, their 30-day mortality rates were only marginally higher than those of non-diabetics, and the difference was not statistically significant. However, the long-term survival data clearly indicate that the presence of diabetes continues to exert a detrimental influence on patient outcomes over time. Long-term survival rates observed in this study show significantly higher survival rates for the non-diabetic group. After 10 years, approximately 80% of non-diabetics were still alive, compared to only 65% of diabetics. At 15 years, 49.98% of diabetics and 66.25% of non-diabetics remained alive. Since the cause of death was not differentiated by cardiac events, it is likely that the reduced survival time in diabetics is largely attributable to non-cardiac comorbidities. Additionally, the 3.5-year higher average age of the diabetic group must be taken into account when interpreting the lower long-term survival. Kaplan–Meier survival curve shows a median survival of 14.54 years for diabetics compared to 19.6 years for non-diabetics. This finding underscores the long-term burden of diabetes on cardiovascular health, even after successful revascularization. Taggart et al. [12] reported a 10-year survival rate of 78.5% for diabetics following MAG, compared to 65.1% in the Essen study. However, the comparability of the patient cohorts is limited, as the Essen patients were on average 2 years older, had a significantly higher proportion of women (28.13% vs. 18.30%), had a higher BMI (29.05 vs. 28.4), and were characterized by much worse NYHA classifications (NYHA stage III-IV: 36% vs. 22.5%). These variables alone are unlikely to explain the full extent of survival differences observed. Several factors contribute to the poorer long-term outcomes in diabetic patients. The higher incidence of comorbidities, including chronic kidney disease and hypertension, likely plays a role. Additionally, the progression of atherosclerosis is often accelerated in diabetics, increasing the likelihood of future cardiovascular events, even after surgical intervention.

This study has several limitations that should be acknowledged. It is a retrospective observational study with data collected prospectively but analyzed retrospectively. A significant limitation is the absence of postoperative angiographic data, which prevents drawing conclusions about graft patency and limits the assessment of the long-term efficacy of MAG. Future studies should incorporate routine postoperative angiography to better evaluate graft success and patency over time. The non-randomized design of the study introduces the possibility of selection bias, as patients were not randomly assigned to receive CABG with MAG. This bias could affect the generalizability of the findings. Future research should aim to minimize this bias through randomized controlled trials or apply methods such as propensity score matching to better control for confounding variables. Additionally, the fact that only 51.95% of diabetics received complete arterial BITA transplantation compared to 67.75% of non-diabetics is a notable limitation, potentially impacting the comparability of outcomes between diabetic and non-diabetic groups. Missing data could not be retrieved retrospectively, and variability in follow-up duration—with long-term data ≥10 years stemming from surgeries conducted between 1999 and 2009—could impact the consistency of outcome interpretation. Although the follow-up rate of 93% for long-term survival is satisfactory, the study lacks information on long-term revascularization needs, occurrences of stroke or kidney failure, postoperative symptom relief, and patients’ subjective quality of life. Including these outcomes in future research could provide a more comprehensive understanding of patient recovery and the overall success of the procedure.

## 5. Conclusions

Diabetes is a significant risk factor influencing surgical outcomes and should be considered in the choice of grafting strategy. It significantly influences the outcomes of patients undergoing CABG with MAG, leading to a higher incidence of certain adverse events and a lower median overall survival compared to non-diabetics. These findings underscore the need for personalized treatment strategies that take into account the unique risks associated with diabetes. Future research should focus on optimizing treatment strategies for diabetic patients undergoing CABG and perioperative management protocols to mitigate risks and improve outcomes for diabetic patients undergoing CABG with MAG.

## Figures and Tables

**Table 1:** Frequency of comorbidities and risk factors.

Condition	Diabetics (*n* = 256)	Non-Diabetics (*n* = 800)	*p*-Value
BMI ≥ 30	93 (36.33)	179 (22.38)	<0.0001
COPD	33/256 (12.89)	84/800 (10.5)	0.29
Peripheral Arterial Disease	51/256 (19.92)	97/800 (12.13)	0.002
Carotid Artery Disease	46/256 (17.97)	109/800 (13.63)	0.09
History of Stroke	17/256 (6.64)	37/800 (4.63)	0.2
Arterial Hypertension	233/256 (91.02)	693/800 (86.63)	0.06
Pulmonary Hypertension	13/256 (5.08)	200/800 (2.5)	0.04
Kidney Disease *	12/240 (4.76)	9/792 (1.14)	0.0004
Active Smoker	103/256 (40.23)	370/800 46.25	0.09
Hyperlipoproteinemia	183/256 (71.48)	598/800 (74.75)	0.3
Familial Predisposition	39/256 (15.23)	194/800 (24.25)	0.003

Data presented as numbers and percentages (%). * Serum creatinine > 2 mg/dL.

**Table 2:** Preoperatively measured left ventricular ejection fraction.

LVEF (%)	Diabetics (*n* = 256)	Non-Diabetics (*n* = 800)	*p*-Value
≥50	174/245 (71.02)	619/770 (80.39)	0.002
30–49	59/245 (24.08)	136/770 (17.66)	0.03
<30	12/245 (4.9)	15/770 (1.95)	0.01

Data presented as numbers and percentages (%).

**Table 3:** Bypass graft characteristics in diabetics and non-diabetics.

Bypass Graft Characteristics	Diabetics (*n* = 256)	Non-Diabetics (*n* = 800)	*p*-Value
BITA	133/256 (51.95)	542/800 (67.75)	<0.0001
BITA + SVG	35/256 (13.67)	144/800 (18)	0.11
ITA + RA	63/256 (24.61)	76/800 (9.5)	<0.0001
ITA + RA + SVG	21/256 (8.2)	25/800 (3.13)	0.0005
BITA + RA	4/256 (1.56)	13/800 (1.63)	0.94
BITA + RA + SVG	0/256 (0)	0/800 (0)	-

Data presented as numbers and percentages (%).

**Table 4:** Hospital stay duration.

Category	Diabetics (*n* = 256)	Non-Diabetics (*n* = 800)	*p*-Value
Total Stay (Days)	13.32 ± 11.4	11.47 ± 6.84	0.002
Postoperative Stay (Days)	9.33 ± 10.68	8.28 ± 6.33	0.05
Intensive Care Stay (Days)	3.15 ± 3.97	2.48 ± 3.61	0.01

**Table 5:** Postoperative adverse events.

Complications	Diabetics (*n* = 256)	Non-Diabetics (*n* = 800)	*p*-Value
Sepsis	5/251 (1.99)	10/793 (1.26)	0.4
Pneumonia	13/251 (5.18)	27/793 (3.4)	0.2
Stroke	5/251 (1.99)	8/793 (1.01)	0.22
PCCS	10/251 (3.98)	5/793 (0.63)	<0.0001
Resuscitation	10/251 (3.98)	22/793 (2.77)	0.33
CVVHD	31/251 (12.35)	41/793 (5.17)	<0.0001
Delirium	39/251 (15.54)	81/793 (10.21)	0.02

Data presented as numbers and percentages (%).

**Table 6:** A 30-day mortality subgroup analysis of diabetes mellitus.

	Diabetics *^t^	Non-Diabetics *^t^	*p*-Value
30-Day Mortality	15/232 (6.47%)	27/753 (3.59%)	0.06
Cardiac	10/15 (66.67%)	12/27 (44.44%)	0.17
Non-Cardiac	3/15 (20.0%)	13/27 (48.15%)	0.07
Unknown	2/15 (13.33%)	2/27 (7.41%)	0.53
	Diabetics *^ie^	Non-Diabetics *^ie^	***p*-Value**
30-Day Mortality	8/200 (4.0%)	15/664 (2.26%)	0.18
Cardiac	6/8 (75.0%)	8/15 (53.33%)	0.31
Non-Cardiac	1/8 (12.5%)	6/15 (40.0%)	0.17
Unknown	1/8 (12.5%)	1/15 (6.67%)	0.64

Data are presented as numbers and percentages (%); *^t^ refers to the total group; *^ie^ refers to isolated and elective CABG—emergency patients and those with concomitant procedures are not included.

**Table 7:** Long-term survival.

Survival Rate	Diabetics *^ie^ (*n* = 200)	Non-diabetics *^ie^ (*n* = 664)
Median Survival (Years)	14.54	19.6
1-Year Survival Rate (%)	93.47	96.38
5-Year Survival Rate (%)	86.08	91.57
10-Year Survival Rate (%)	65.1	79.73
15-Year Survival Rate (%)	49.98	66.25
20-Year Survival Rate (%)	35.08	49.61

*^ie^ refers to isolated and elective CABG—emergency cases and those with concomitant procedures are not included.

## Data Availability

The original contributions presented in this study are included in the article material. Further inquiries can be directed to the corresponding author.
